# Evolutionary analysis of the Chikungunya virus epidemic in Mexico reveals intra-host mutational hotspots in the E1 protein

**DOI:** 10.1371/journal.pone.0209292

**Published:** 2018-12-14

**Authors:** José Esteban Muñoz-Medina, Miguel Antonio Garcia-Knight, Alejandro Sanchez-Flores, Irma Eloísa Monroy-Muñoz, Ricardo Grande, Joakim Esbjörnsson, Clara Esperanza Santacruz-Tinoco, César Raúl González-Bonilla

**Affiliations:** 1 Laboratorio Central de Epidemiología, Centro Médico Nacional “La Raza”, Instituto Mexicano del Seguro Social, Mexico City, Mexico; 2 Centro de Investigaciones en Ciencias Microbiológicas, Instituto de Ciencias, Benemérita Universidad Autónoma de Puebla, Puebla, Mexico; 3 Unidad Universitaria de Secuenciación Masiva y Bioinformática, Instituto de Biotecnología, Universidad Nacional Autónoma de México, Cuernavaca, Morelos, Mexico; 4 Laboratorio de Genómica, Departamento de Genética y Genómica Humana, Instituto Nacional de Perinatología “Isidro Espinosa de los Reyes”, Mexico City, México; 5 Systems Virology, Department of Laboratory Medicine, Lund University, Lund, Sweden; 6 NDM Research Building, Nuffield Department Medicine, University of Oxford, Oxford, United Kingdom; 7 División de Laboratorios de Vigilancia e Investigación Epidemiológica, Instituto Mexicano del Seguro Social, Mexico City, Mexico; Institut Pasteur Montevideo, URUGUAY

## Abstract

The epidemic potential of Chikungunya virus (CHIKV) was recently made evident by its introduction and rapid expansion in the Caribbean and the Americas. We sought to gain a detailed understanding of the dynamics of the epidemic in Mexico, the country with the highest number of confirmed CHIKV cases in the Americas, and to characterise viral evolution at the population and intra-host level. Analysis of the spatiotemporal distribution of 2,739 diagnosed cases in Mexico from December 2014 to December 2015 showed a rapid nationwide expansion of the epidemic with focalisation in the South West of the country. We sequenced the envelope glycoprotein 1 gene (E1) from 25 patients using the Illumina MiSeq platform and report synonymous and non-synonymous consensus mutations. Bayesian phylogenetic analysis using 249 Asian lineage E1 sequences gave updated estimates of nucleotide substitution rates for E1 and time to most recent common ancestor of major lineages. The analysis indicates phylogenetically-related emergent Latin American clusters in South Western Mexico, Nicaragua and Honduras and transmission of American strains in the Pacific islands. Detailed analysis showed that intra-host changes in E1 mainly occurred in two variable regions (E1:189–220 and E1:349–358) in domains II and III, respectively, in residues involved in inter and intra-envelope spike interactions. At the population level, this study sheds light on the introduction and evolutionary dynamics of CHIKV in the Americas. At the intra-host level, this study identifies mutational hotspots of the E1 protein with implications for understanding the relationship between the CHIKV quasispecies, viral fitness and pathogenesis.

## Introduction

Chikungunya fever is an infectious disease caused by the Chikungunya virus (CHIKV), an arbovirus of the *Togaviridae* family with a widespread and expanding global distribution. First identified in Tanzania in 1952, CHIKV is principally transmitted, in urban transmission cycles, by *Aedes aegypti* mosquitoes that mainly inhabit tropical and subtropical regions[1,2]. CHIKV has a 11.8kb positive sense RNA genome that encodes four non-structural proteins (nsP1-4) and three main structural proteins (capsid and the spike forming envelope glycoproteins [E1&2])[3]. Though rarely fatal, CHIKV has a high attack rate and infection can lead to high fever, headache, rash and characteristic severe debilitating arthralgia which can persist. To date, no specific prophylactic or therapeutic interventions exist.

Phylogenetic analyses of CHIKV have identified three main lineages: the West African, East Central South African (ECSA) and Asian linage[4]. In 2004, an epidemic of the ECSA lineage re-emerged in Africa and the Indian Ocean and evolved into a novel Indian Ocean Lineage (IOL)[5,6]. Adaptive mutations (e.g. E1:A226V[7] & E2: L210Q[8]) facilitating transmission by *Ae*. *albopictus* emerged in IOL strains and significant outbreaks occurred in India, South East Asia and Italy. The first autochthonous cases of CHIKV in the Western Hemisphere were reported in Martinique in December 2013; rapid epidemic expansion followed throughout the Caribbean and Latin America leading to over 30,000 laboratory confirmed cases by April 2015[9]. The epidemic in the Americas is derived from the re-emergent Asian lineage[10–12], (though ECSA lineage infections lacking E1:A226V have been identified in Brazil[11]); a novel Caribbean genotype has been described in isolates from across the Americas containing a transmission-facilitating 3’ untranslated region duplication[13].

The first autochthonous CHIKV case in Mexico was reported in October 2014 in the southern border state of Chiapas[12]. By November, cases had been confirmed in nine states and the Mexican Institute of Social Security (IMSS, “Instituto Mexicano del Seguro Social”), which provides health care to ~60% of the population[14], implemented a confirmatory diagnosis algorithm for CHIKV following guidelines set by the National Institute of Diagnosis and Reference (InDRE, “Instituto de Diagnóstico y Referencia Epidemiológicos”). Here we analyse the cumulative confirmed cases reported within the IMSS by state from December 2014 to December 2015. We also characterized temporally and geographically representative E1 gene sequences and present an up-to-date phylogenetic analysis of CHIKV lineages, with a focus on the Asian lineage epidemic in the Americas and the Caribbean. Finally, we explore the differing degrees of E1 protein diversity in the viral quasispecies within patients and identify hotspots of intra-patient mutation.

## Results

### Cumulative cases of CHIKV in Mexico

Out of 5,266 patient serum samples received by the LCE between December 2014 and December 2015, 2,739 samples (52.0%) were CHIKV positive. Univariate analysis indicated that age and geographical region, but not gender, was associated with CHIKV infection. 18–29 years made up the largest age category with positive cases (22.3%) and Mexican states in the south of the country reported most cases (78.4%; **S1 Table**).

**Fig 1:**
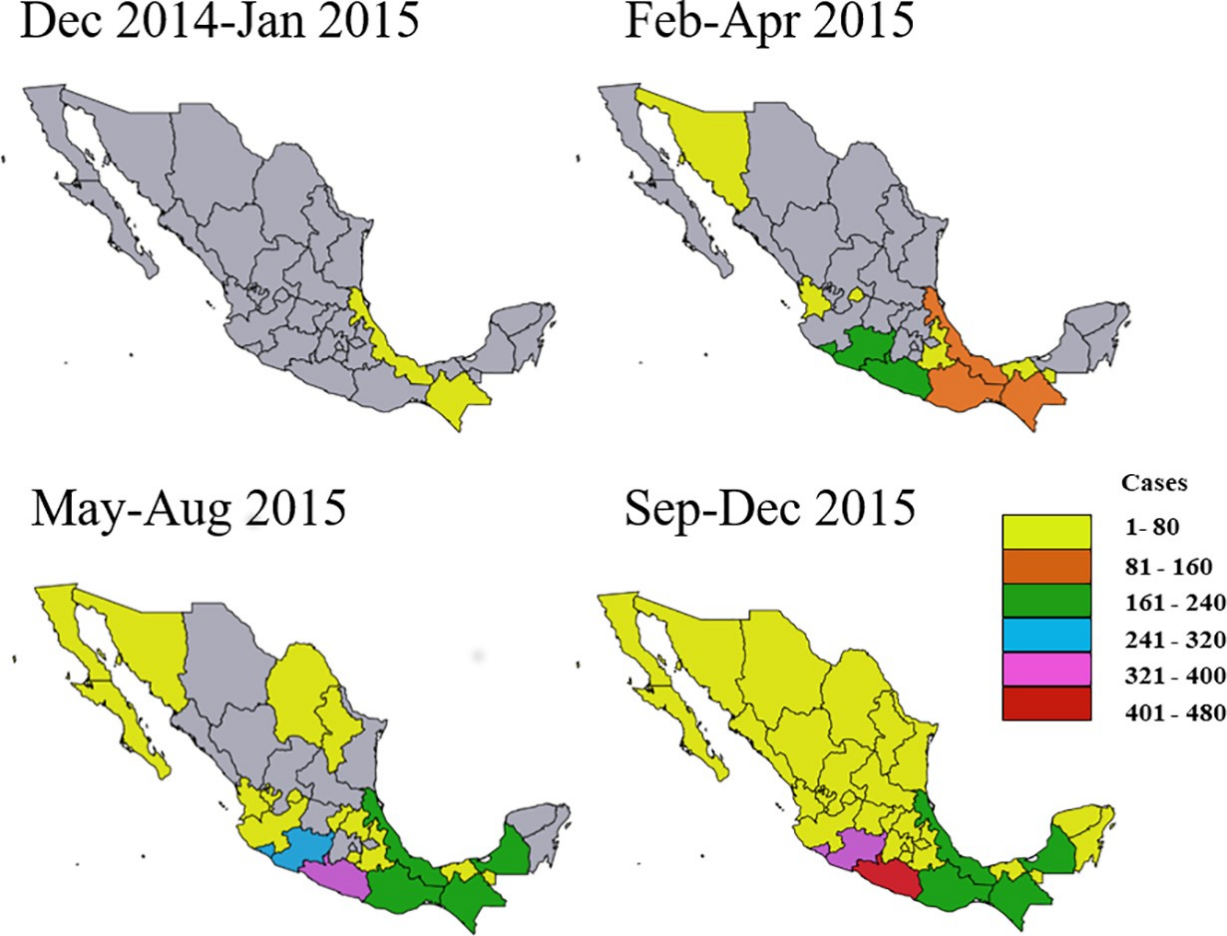
Accumulation of confirmed CHIKV cases per state over time. Political maps of Mexico with state borders shown. Confirmed cases through molecular diagnosis were from patients who attended any of the 35 IMSS clinical centres nationwide. Grey indicates that no cases were reported.

Analysis of the cumulative positive cases in Mexico indicated an initial appearance of the disease in the states of Chiapas and Veracruz from December 2014 to January 2015, followed by cases in states on the Pacific coast including Guerrero, Oaxaca, Colima and the northern state of Sonora (**Fig 1**). By mid-2015, infections were detected in central and northern states and in the Yucatan peninsula (**Fig 1**) and by December 2015, IMSS clinical sites from all states in Mexico reported confirmed cases.

Specimens from 500 patients sampled from January-November 2014 who met the definition for suspected DENV infection but who were found to be DENV-negative, were analysed to assess CHIKV infection. No CHIKV-positive samples were identified prior to December 2014.

### Novel nonsynonymous mutation in a CHIKV E1 consensus sequence

To characterise the CHIKV strains circulating in Mexico during the epidemic, we determined the consensus sequence of the E1 glycoprotein-encoding gene from 25 CHIKV-infected patients (**S2 Table**) from five Mexican states (five from each state) in the Central/southern region which had most confirmed CHIKV cases by August 2015. Two partial amplicons from the E1 gene were generated (COF1 and CIF2) and deep sequenced. The most abundant sequences from each were overlapped and used to reconstruct a 1044bp region from the E1 gene (E1:270–1314).

Amongst all patient-derived consensus sequences, we observed a total of nine nucleotide substitutions resulting in two nonsynonymous mutations (**Table 1**). Most of the observed nucleotide substitutions occurred in separate specimens except for A741G and G855A mutations which were observed in six and two specimens, respectively. The nonsynonymous T207M and V291I mutations were observed in specimens from patients from the Pacific states of Colima and Guerrero, respectively.

**Table 1:** Mutations in partial CHIKV E1 consensus sequences from Mexican patients.

Nucleotide change	Codon position	AA change	Genomic position*	Sample
C384T	3	Syn>S	10,377	Chiapas_8
T516A	3	Syn>P	10,509	Veracruz_1
C620T	2	T207M	10,613	Colima_13
A741G	3	Syn>R	10,738	Guerrero_3, Guerrero_9, Guerrero_15, Guerrero_16, Guerrero_18 & Colima_23
G855A	3	Syn>A	10,848	Guerrero_9 & Guerrero_15
G871A	1	V291I	10,864	Guerrero_15
T1054C	1	Syn>L	11,047	Guerrero_15
A1200G	3	Syn>Q	11,193	Colima_2
T1248C	3	Syn>G	11,241	Veracruz_20

*S27 genome; AA, amino acid; Syn, synonymous

### Evolution of the major CHIKV lineages

**Fig 2:**
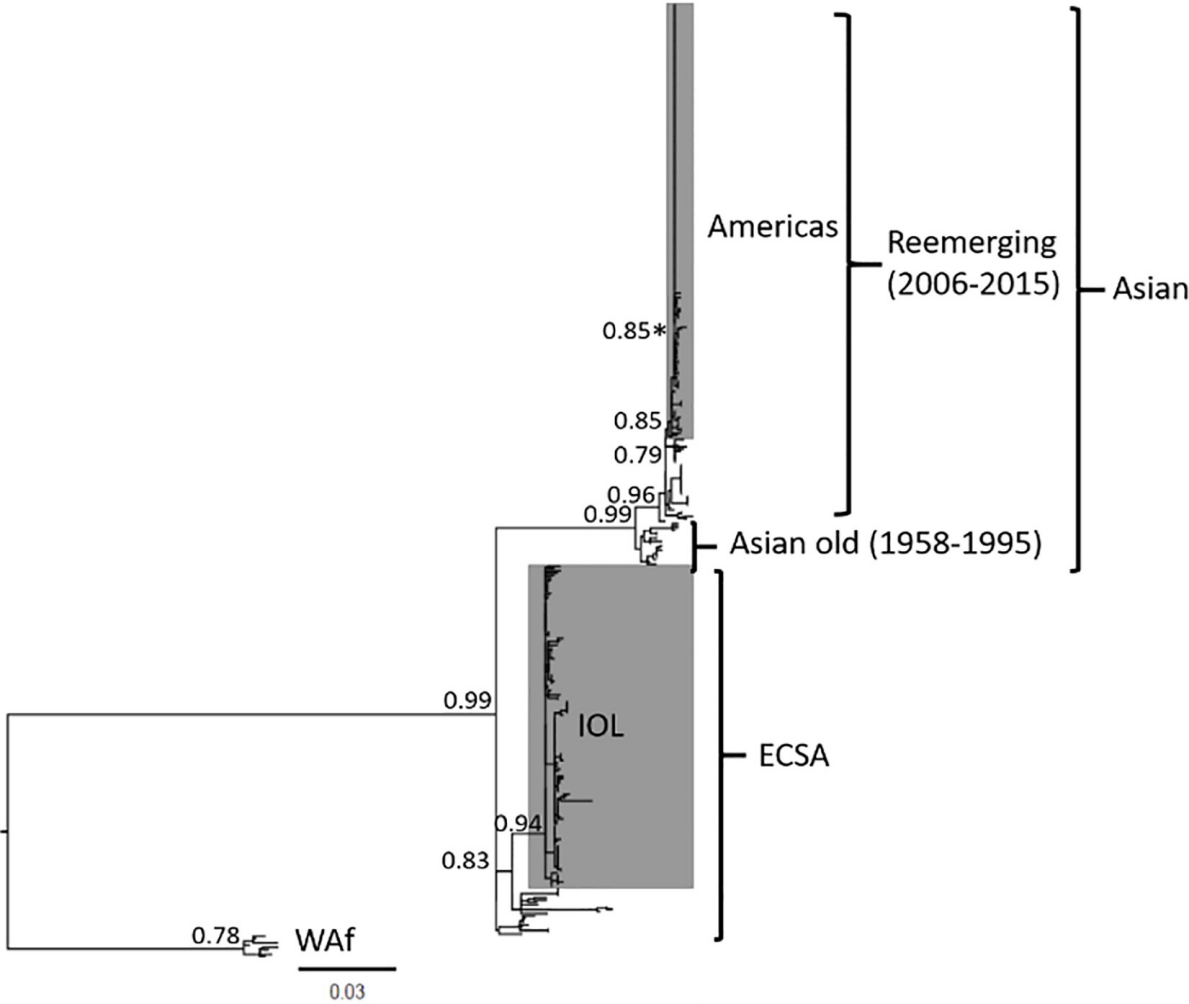
Maximum likelihood phylogenetic analysis of partial CHIKV E1 gene sequences. A phylogeny of 420 CHIKV strains with an O’nyong-nyong strain used as an outgroup (not shown) built using the General Time Reversible substitution model with a gamma distributed rate variation among sites. Statistical support for major tree nodes is shown as aLRT scores. Epidemic linages are shown in grey and a statistically supported cluster of 6 Mexican strains is marked with an asterisk. The scale bar represents nucleotide substitutions per site. ECSA, East-Central-South Africa; IOL, Indian Ocean Lineage; WAf, West African.

To assess the evolutionary relationship between the 25 Mexican strains and CHIKV strains sampled globally, and to provide an updated evaluation of evolution within the Asian lineage using all available E1 sequences, we aligned our sequences with 395 CHIKV strains. Our initial maximum likelihood analysis did not resolve the ECSA lineage as being monophyletic, as has been shown in analyses using whole genomes [15]. However, the use of a starting tree resulted in a maximum likelihood phylogeny that resolved the three major CHIKV lineages (Asian, ECSA and WAf) with good branch support for all lineages including the IOL and American and Caribbean epidemic sublineage (**Fig 2**). As expected, the 25 Mexican sequences all clustered with the epidemic Asian lineage strains from the Americas.

Next, we analysed the major CHIKV lineages shown in **Fig 2** separately using a Bayesian coalescence approach to estimate the evolutionary rate and the time to the most recent common ancestor (tMRCA) of each lineage. We initially assessed the evolutionary signal of the alignments using root-to-tip regression estimates (**S1 Fig**). We then analysed the Asian all, Asian old, non-IOL ECSA, IOL and WAf datasets, which had strong evolutionary signals (*R*^*2*^ >0.63 for all datasets); the Asian sequences sampled post 1995 had a poor evolutionary signal (*R*^*2*^ = 0.15) and were not analysed (**S1 Fig**).

The evolutionary rate of the entire Asian lineage was similar to the rate of the Asian old dataset (6.71E-4 [95% HPD: 4.92E-4, 8.51E-4] vs 6.52E-4 [95% HPD: 3.01E-4, 9.91E-4] nucleotide substitutions/site/year), indicating that the recent epidemics strains evolve at a similar rate to older Asian lineage strains (**Table 2**). The highest evolutionary rate estimate was for the IOL lineage (15.44E-4 [95% HPD: 11.17E-4, 20.28E-4] substitutions/site/year) which was over two-fold higher than the non-IOL ECSA lineage (6.04E-4 [95% HPD: 3.17E-4, 9.40E-4] substitutions/site/year). The lowest mean evolutionary rate estimate was observed for the WAf lineage (4.93E-4 [95% HPD 1.93E-4, 9.38E-4] substitutions/site/year).

**Table 2:** Rate of nucleotide substitutions and tMRCAs of mayor CHIKV lineages.

Dataset	Evolutionary rate Mean nucleotide substitutions/site/year (95% HPD interval)	tMRCA (root height 95% HPD interval)
Asian all	6.71E-4 (4.92E-4-8.51E-4)	07/1957 (01/1955-07/1958)
Asian old	6.52E-4 (3.01E-4-9.91E-4)	08/1957 (12/1954-07/1958)
ECSA	6.04E-4 (3.17E-4-9.40E-4)	11/1947 (04/1937-02/1953)
IOL	15.44E-4 (11.17E-4-20.28E-4)	11/2003 (09/2002-07/2004)
WAf	4.93E-4 (1.93E-4-9.38E-4)	05/1961 (06/1954-07/1964)

### Phylogeny of the Asian lineage

**Fig 3:**
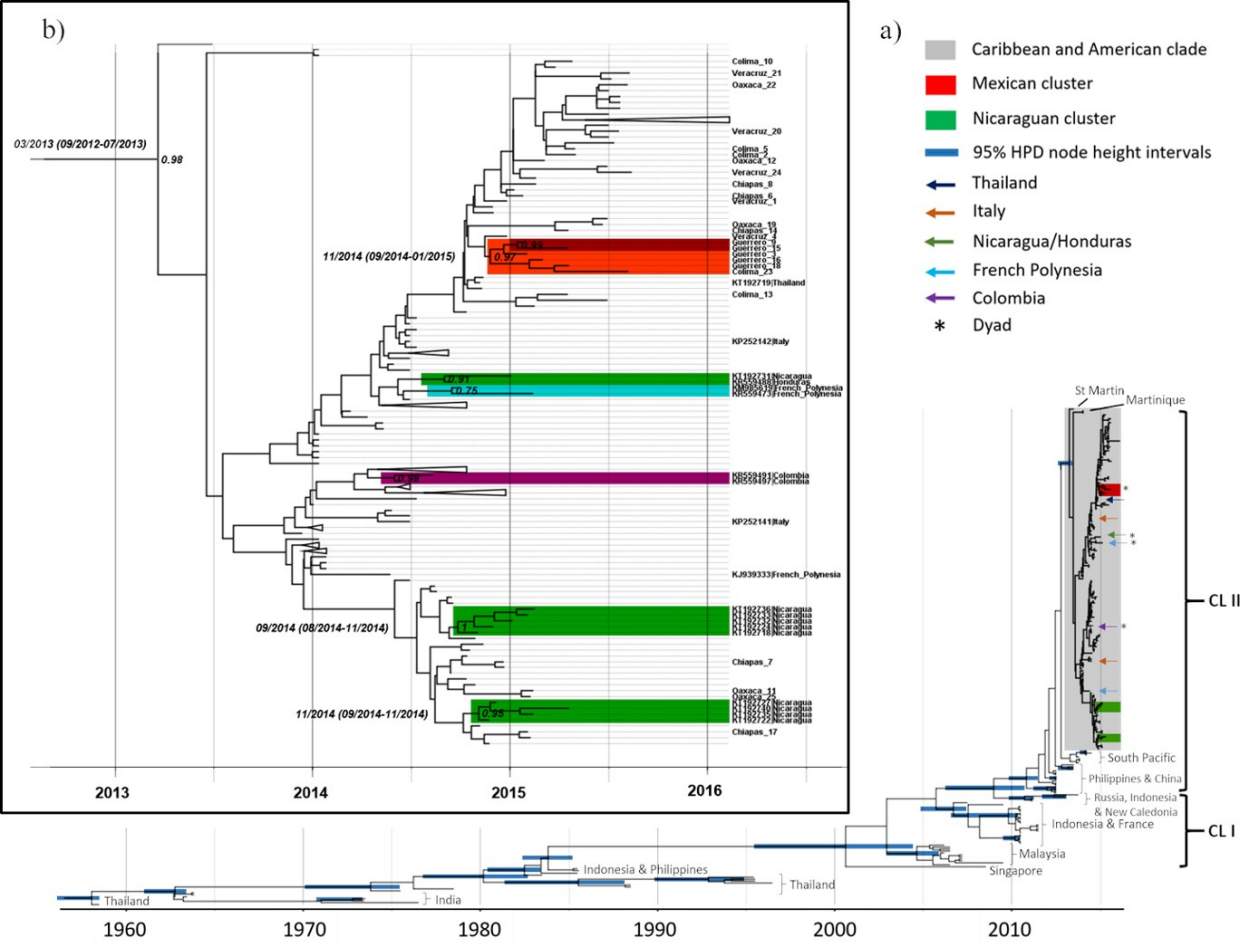
Bayesian phylogenetic analysis of partial CHIKV E1 gene sequences. (A) Bayesian MCC tree of 249 Asian lineage strains calibrated to the sampling date and built using the HKY+G nucleotide substitution model, a lognormal relaxed clock and a Bayesian Skygrid coalescent model. Nodes with posterior probabilities (clade-credibility values) ≥0.9 have 95% HPD intervals for node heights shown. The two main epidemic Asian lineage clades (CL) are also shown. (B) A subtree of the American and Caribbean sublineage showing posterior probabilities and calibrated tMRCA and 95% HPD intervals for statistically supported monophyletic groups. For clarity, node bars with 95% HPD intervals are not shown, only relevant strains are labelled, and the colour code is as in section a).

To assess the evolution of the Asian lineage in detail, we analysed the MCC tree of all Asian lineage sequences (**Fig 3A**). Our analysis largely recapitulates the evolutionary relationships observed in studies using whole genomes sequences[4,15,16]: i) emergence of the Asian lineage in the late 1950s (07/1957 [95% HPD 01/1955-07/1958]; **Table 2**); ii) early evolution of Thai and Indian clades and probable extinction of the India clade; iii) introduction of a Thai lineage into Indonesia and the Philippines in the early 1980s (01/1980 [95%HPD: 11/1976-09/1982]); and iv) endemic transmission prior to the emergence of epidemic lineages from South East Asia and Oceania which derived from a common ancestor in the early 2000s (03/2000 [95% HPD: 04/2004-08/1995]).

Studies using whole genomes have additionally identified two major recent monophyletic epidemic clades[15,17]. Our phylogeny did not resolve the monophyly of clade I (**Fig 3A**). However, clade II was estimated to have originated late in 2010 (09/2010 [95% HPD: 06/2009-10/2011]) in South East Asia where it spread to Pacific Island nations such as Micronesia, Tonga and American Samoa. These sequences are the most closely related to the epidemic sublineage in the Caribbean and Americas, which arose at the start of 2013 (03/2013 [95% HPD: 09/2012-07/2013]; **Fig 3B**). Within this sublineage, a well-supported (posterior probability [PP] > 0.9) Mexican cluster (all five specimens form the state of Guerrero and one from Colima [Colima_23]) and two Nicaraguan (accession numbers: KT192722, KT192727, KT192735 and KT192740; and KT192718, KT192724, KT192732, KT192733, KT192736) clusters were found, emerging in late 2014 (11/2014 [95% HPD: 09/2014-01/2015], 11/2014 [95% HPD: 09/2014-11/2014] and 09/2014 [95% HPD: 08/2014-11/2014], respectively; **Fig 3B**). Sequences forming dyad clusters were also detected from Mexico (Guerrero_9 and Guerrero_15), Nicaragua/Honduras (KT192731 and KR559488) Colombia/Unknown origin (KR559491 and KR559497) and French Polynesia (KM985619 and KR559473). Interestingly, the former dyad (PP = 0.75) and a third sequence from French Polynesia (KJ939333) suggests possible local transmission of the American and Caribbean sublineage in the Pacific islands. By contrast, the two sequences from Italy (KP252141 and KP252142) and one from Thailand (KT192719), are likely indicative of traveller-associated infection.

### Intra-patient diversity of CHIKV

**Fig 4:**
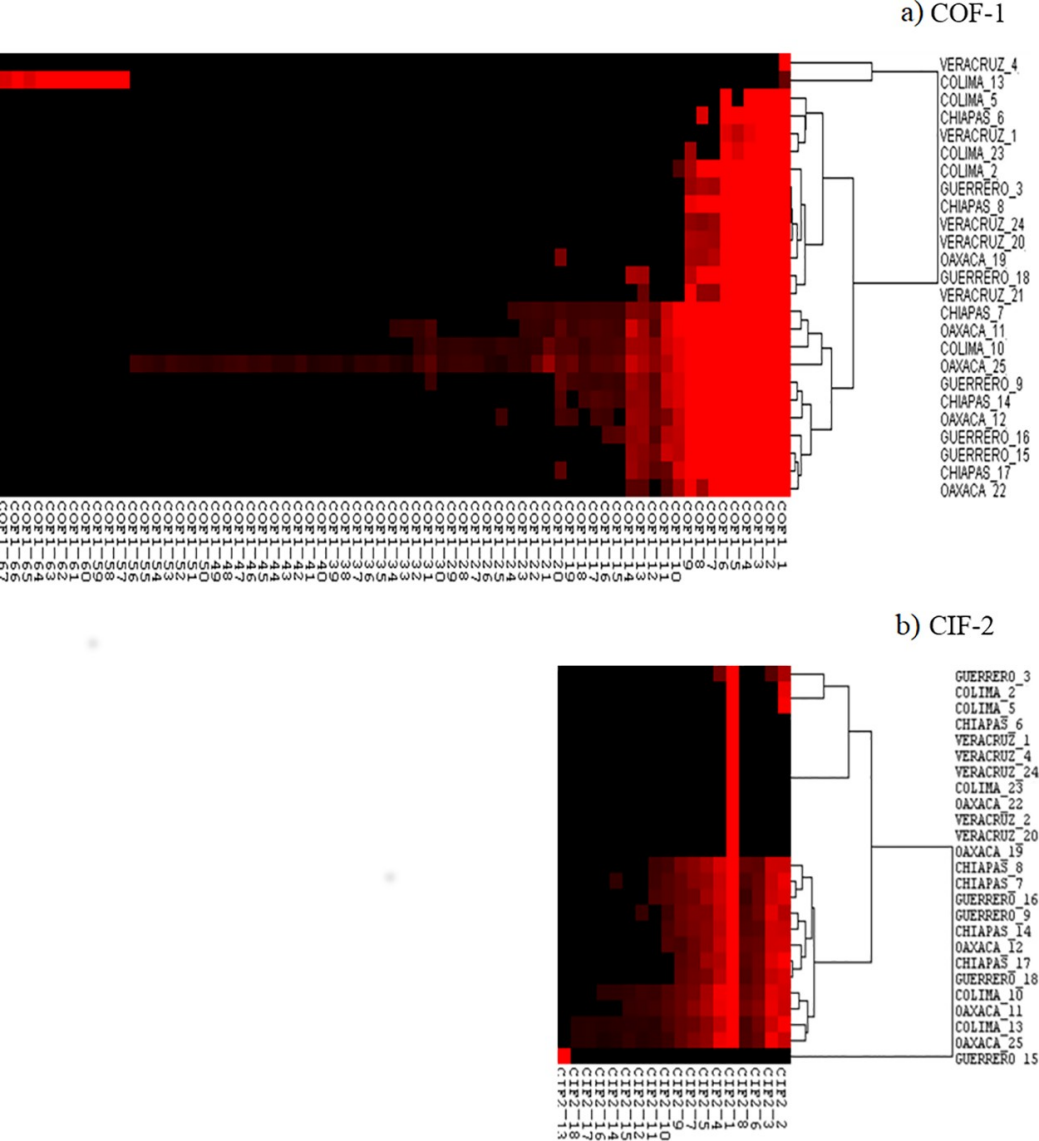
Abundance of viral haplotypes in two E1 gene amplicons. Heat maps indicating the relative abundance (red colour intensity) of distinct amino acid viral haplotypes following deep sequencing of two amplicons COF 1 (A) and CIF 2 (B) that span the near full-length CHIKV E1 gene in 25 Mexican patients. Dendrograms represent clustering of patients according to haplotype diversity.

The use of next generation sequencing allowed us to analyse intra-patient variability in the reconstructed E1 gene amplicons (COF1 and CIF2; sequencing yields shown in **S3 Table**). After clustering the nucleotide sequences with a minimum 50 amplicons, we clustered the translated products for each nucleotide cluster with 100% identity, obtaining 67 amino acid sequence haplotypes for COF1 and 18 amino acid sequence haplotypes for CIF2. The relative abundance of each haplotype is shown in **Fig 4**. A wide degree of heterogeneity in the intra-patient diversity of viral haplotypes was seen, and the number of variants present in COF1 and CIF2 correlated significantly amongst patients (**S2 Fig**). Variant number ranged from the presence of a single variant (COF1_1 in patient Veracruz_4, CIF2_1 in eight patients and CIF2_13 in Guerrero_15;) to 56 COF1 and 17 CIF2 variants within a single patient (Oaxaca_25) with diverse degrees of abundance (**Fig 4**). We also observed the same dominant haplotypes in both amplicons (COF1_1 and CIF2_1) in all patients except for Colima_13 and Guerrero_15 (**Fig 4**), where separate nonsynonymous mutations in the consensus sequence were present at a frequency >50% (**Table 1 and S4 Table**). In COF1, six haplotypes (COF1_1–6) were highly abundant in most patients, whereas in CIF2, patients either had nine or more variant haplotypes (N = 12) or 1–4 variants (N = 13).

**Fig 5:**
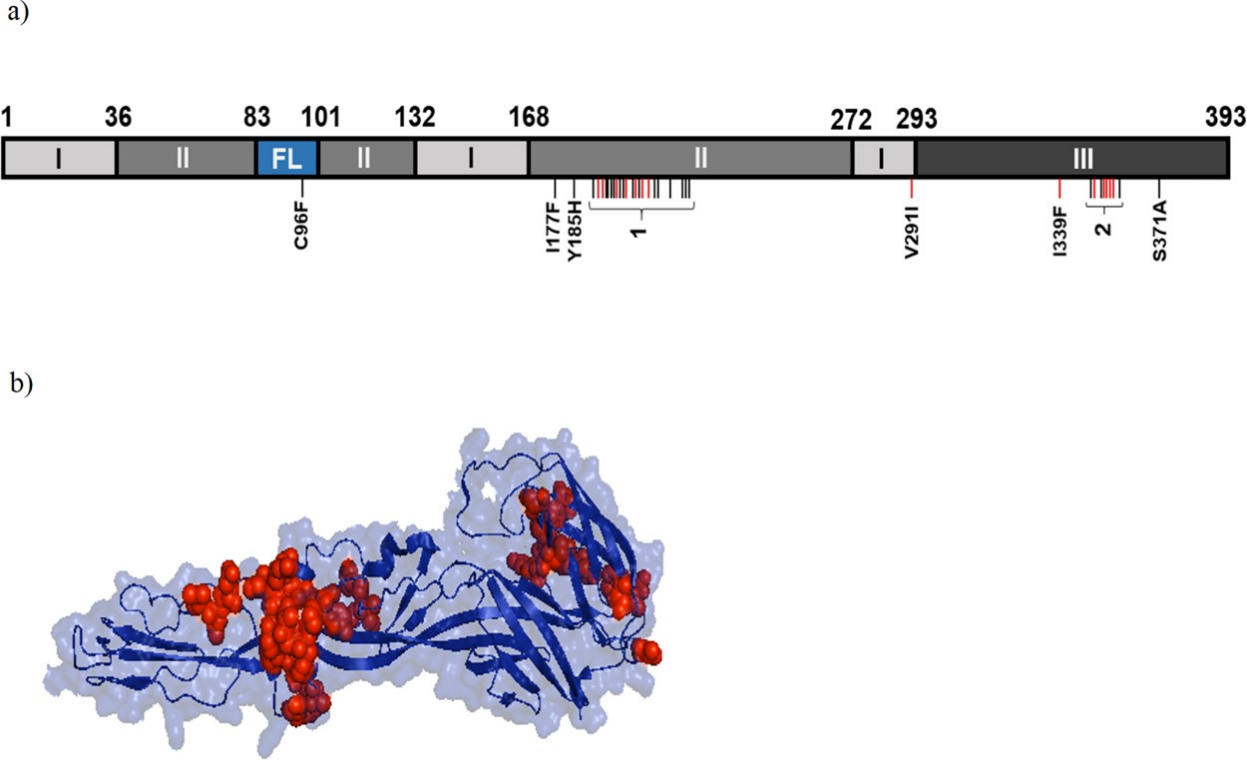
Sites of intrapatient mutations in CHIKV E1 protein. (A)Linear diagram of CHIKV E1 protein (adapted from Voss et al[18] indicating protein domains, amino acid positions (top) and the positions of amino acid mutations (vertical bars) including two mutational hotspots (1 & 2) with the following mutations in order of location: 1 = Y189D, P191T/A/H/R/S, F192V/C/L/S/Y/I, G193D, A194E/G, A195R, R196S/G, P197Q/T, G198V, Q199H/K, F200V/I/C/L, D202E, I203S/T, Q204L/H/P, S205R/G, T207M, E209A, S210R, Y214X, Q218P/H, L219R, V220G; and 2 = N349K, S350A, L352W, Q353K/P, I354S/L/T, S355A/P, F356C/L/S, T358P). Red bars indicate mutations with frequencies >0.01 and that were present in >50% of patients or mutations present in the consensus sequence of a single patient. FL, fusion loop. (B) Structural model of native CHIKV E1 protein indicating the locations of intrapatient point mutations (red).

Analysis of the location of E1 amino acid mutations amongst the intra-patient quasispecies indicated that most variation occurred within domains II and III (**Fig 5**). Low frequency (0.3%) C96F mutations in the fusion loop were also observed in four patients (**Fig 5A and S4 Table**) and the dominant V291I mutation in patient Guerrero_15 mapped to domain I (**Fig 5A**). Of note, variation was centred around two regions located between E1 residues 189–220 (hotspot one; 22/38 variation sites) and 349–358 (hotspot two; 8/38 variation sites; **Fig 5A**) in domain II and III, respectively. Nine and 13 mutations within hotspots one and two, respectively, occurred in over a third of patients and at average frequencies >1% **(S4 Table)**. These two regions include residues involved in E1-E1 inter-spike interactions (191–194, 351, 353 and 355) and E2-E1 intra-spike interaction (196–199) as well as residues highly conserved amongst alphaviruses: 193, 198, 202, 204 and 356[18].

## Discussion

We sought to gain insights into CHIKV evolution by analysing the emergent epidemic in Mexico, where over 11,000 confirmed cases were reported in 2015, the highest number in any country that year [19]. As seen in other non-endemic countries, with the exception of Panama[20], CHIKV spread rapidly to tropical regions nationwide. The endemicity of the primary vector *Ae*. *aegytpi* and an immunologically naïve population were central facilitators of the epidemic; however specific factors leading to the focalisation of cases in the South of the country, particularly along the Pacific Ocean, merit attention. Of note, viruses from this region harboured non-synonymous mutations and formed an emergent cluster, suggesting localised evolution of CHIKV in Mexico. Interestingly, the number of Zika virus (ZIKV) cases during the 2016 epidemic in Mexico was also highest in the Southern states of Guerrero, Veracruz and Yucatán[21]. Striking similarities in transmission dynamics following CHIKV and ZIKV epidemics in the Americas have been observed[22]; our data suggest that these similarities are also apparent within Mexico and this may have implications for future arboviral threats as such as Yellow Fever, Mayaro and Oropuche viruses.

Local CHIKV transmission was first detected in Mexico in October 2014 amid reports of cases in Guatemala and the United States[23]. Similarly to Brazil[11], our analysis supports the introduction of CHIKV shortly before the first autochthonous diagnosed cases, as we found no evidence of CHIKV infection in 500 febrile DENV-negative patients sampled from across the country between January and October 2014. In addition, the tMRCA for the observed Mexican cluster was estimated for November 2014 (September 2014- January 2015 95% HPD). By contrast, ZIKV was reported to be present in Mexican patients 3–10 months prior to the first reports of imported cases[24] and to have circulated in Brazil up to 12 months prior to its detection[25]. We suggest that knowledge of ongoing CHIKV transmission in the region and the high rate of symptomatic infections contributed to the early detection of the first cases in Mexico. Active surveillance of emergent arboviral threats, particularly of those with high numbers of asymptomatic cases, is therefore critical for early containment efforts.

To assess the evolutionary dynamics of major CHIKV lineages in Mexico and worldwide, we analysed E1 gene sequences. A comparison of phylogenetic trees inferred through ML analyses using whole CHIKV genomes and E1 gene sequences, indicated that the former do not adequately resolve the ECSA lineage[4]. However, the use of a starting tree in our ML analysis enabled us to resolve all major lineages, including the ECSA lineage. In addition, separate alignments of major lineages indicated robust phylogenetic signals for the datasets analysed. Importantly, our approach captured diversity from Asian lineage viruses lacking whole genome sequences. Our tMRCA estimates for the Asian, IOL and WAf lineages were similar to studies analysing the whole CHIKV genome[4,15,26]. However, our estimates for the ECSA lineage were 10 to 35 years more recent, reflecting the complex phylogenetic structure of these sequences and the limitations of analyses focused on E1 gene sequences for this lineage[15]. In accordance with a detailed phylogeographic study[17], we estimated, albeit with wider 95% HPD values, that the American and Caribbean sublineage evolved around March 2013 from closely related South Pacific strains. How CHIKV and ZIKV were initially introduced into the Caribbean and Atlantic coast of America, respectively, from Oceania or South-East Asia without apparent prior transmission to the Western coast of the Americas remains unclear. Identifying the ongoing transmission routes of American lineage CHIKV to Pacific Islands, such as French Polynesia, may provide clues, particularly as these cases appear to be forming epidemiologically linked clusters. By contrast, likely imported cases to Italy and Thailand, seem unrelated to recently reported outbreaks[27]. Our result also suggest the presence of emerging clusters in Mexico and Nicaragua and epidemiologically-related transmission between Nicaragua and Honduras which, with other emerging clusters in the region, warrant surveillance, in particular when assessing sylvatic transmission emergence in the Americas[28].

Our evolutionary rate estimates amongst Asian datasets (**Table 1)** were similar to what was reported for E1 by Sahadeo *et al*[17] (6.0 E-4 [95% HPD: 4.0–8.0 E-4] nucleotide substitution/site/year) and higher than what has been reported for whole genomes[4,11,15]. In accordance with Chen *et al*[15], the evolutionary rate of the IOL lineage was over twice that of the ECSA and other lineages -with non-overlapping 95% HPD values- though our estimates were an order of magnitude higher than this report, which was based on the whole genome. The convergent evolution of the adaptive E1:A226V mutation in different IOL clusters[7] likely contributed to the increased the rate of evolution in E1. More generally, the high evolutionary rate estimates of the IOL lineage may result from frequent sampling during the epidemic and inclusion of variants not purged by purifying selection[29], though this effect seems to be less marked in the American and Caribbean sublineage in our study but not others[17].

As expected for an arbovirus where evolution is constrained due to replication in dual hosts[30], variation in the consensus sequences of the E1 gene from 25 patients was limited. We found an undescribed T207M mutation in a single individual that maps to domain II and lies adjacent to an arginine residue involved in E1-E1 inter-spike contacts[18]. The replacement of a polar amino acid with a hydrophobic residue indicates possible structural changes that should be characterised functionally. We also observed a V291I mutation that was seen in five patients in an outbreak of the IOL lineage in Kerala, India between 2009 and 2013[31] and in one patient from Nicaragua sampled in December 2014[32]. V291I maps to domain I and the outer surface of a E1 trimer were it has been suggested to have a limited role in modulating infectivity[31].

The massive parallel sequencing of samples also enabled us to analyse viral diversity in each patient. To assess the entire haplotype of each E1 amplicon (i.e. mutations present in *cis*) we only included overlapping paired end reads that reconstructed the amplicons and had a sequencing depth of at least 50 overlapping reads. We used this approach to reduce the inclusion of sequencing errors or artefacts, allowing us to explore a real representation of the diversity for each patient. Strikingly, a marked heterogeneity in the level of haplotype diversity at the amino acid level was seen in each patient, with levels consistent over both COF and CIF amplicons. The diversity of viral quasispecies is a critical component of viral fitness[33], as demonstrated by reduced fitness and pathogenicity of CHIKV replication fidelity variants[34,35]. Whether intrahost diversity relates to the clinical outcome of CHIKV and other arboviruses, such as dengue[36], is a key question. Although our study did not focus on clinical outcomes, most of the primary symptoms reported by patients analysed by sequencing (fever, arthralgia, myalgia and / or headache) were similar to those observed by authors in Mexico and the Island of Reunion [37–38], though we observed a greater proportion of arthritis and less haemorrhagic cases **(S6 Table)**. In addition, a similar proportion of patients showed high levels of haplotype diversity as those reported to have persistent clinical symptoms in the Americas (~ 50%)[39]. Therefore, in accordance with the variation in clinical outcomes in acute and persistent stages of infection, we agree with the conclusions of previous reports[40] that the association between interpatient viral diversity and clinic outcome warrants further detailed studies.

Of note, the number of non-synonymous intra-host variants observed within the E1 coding sequence was higher than those reported previously in studies assessing the whole CHIKV genome[13,17]. The reasons for this discrepancy may be due to the sequencing depth obtained from analysing E1 sequences alone. We suggest that the inclusion of processing artefacts in our analysis (e.g. PCR-generated mutations) is unlikely. For instance, the presence of the same variant in numerous patients and the non-random distribution of variants in discrete mutational hotspots suggest natural variation. Furthermore, we only report minority variants with frequencies >0.1% that represent reads with ≥50 identical nucleotide sequences after clustering at nucleotide level. Individually, most variants occurred at frequencies <10%, suggesting reduced fitness in comparison to the consensus sequence. Interestingly, similar frequencies of minority variants were observed across patients, suggesting a possible evolutionary advantage to their maintenance in the viral swarm[41]. Alternatively, similar pathways may be used by CHIKV quasispecies to explore sequence space. We also observed a low frequency (0.2%) stop codon mutation (Y214X) in two patients, which may indicate the maintenance of partial or defective genomes[42], with possible fitness effects. Stop codon mutations leading to deletions have similarly been reported in the Venezuelan Equine Encephalitis virus 6K protein [43]. The non-random distribution of intra-host mutations in E1 could be driven by several mechanisms. *Cis* elements that alter template structure and reverse transcriptase fidelity in specific regions of envelope proteins have been demonstrated for HIV[44]. Alternatively, these sites may be under low selective constraints and be influenced by neutral or positive selection, possibly through immune-selection pressure. To our knowledge, no neutralizing antibodies elicited against CHIKV have been shown to target residues between E1 189–220 and 349–358[45–48].

To conclude, we report localised CHIKV transmission and evolution in South West Mexico that gave rise to an emergent Mexican cluster which evolved shortly after the introduction of the Asian lineage of CHIKV into the country. Crucially, the characterisation of the intra-host mutant swarms indicates high levels of E1 protein diversity centred in two previously undescribed mutational hotspots in domains II and III which is common to numerous patients. Whether this variation has a role in determining viral fitness during infection should be further explored mechanistically.

## Methods

### Ethics statement

Human serum specimens were an excess of samples collected during routine passive surveillance activities of the Central Laboratory for Epidemiology (LCE, “Laboratorio Central de Epidemiologia”), Instituto Mexicano del Seguro Social in Mexico City. All specimens were de-linked from any personal identifiers prior to the commencement of the study.

The Study was approved by the Ethics and the Research Committees of the National Committee of Scientific Research of the Instituto Mexicano del Seguro Social with the registration number R-2015-785-096

### Study design

From December 2014 to December 2015, serum samples from all suspect CHIKV cases detected through passive surveillance in 35 IMSS medical centres nationwide (located in 32 Mexican states) were submitted for CHIKV confirmatory diagnosis to the Central Laboratory for Epidemiology (LCE, “Laboratorio Central de Epidemiologia”), IMSS in Mexico City. In accordance with national guidelines, suspect case definition included febrile illness with poly-arthralgia or acute arthritis, plus living in or travelled to, within two weeks of fever onset, an area endemic for *Ae*. *aegypti* or *Ae*. *albopictus* with confirmed CHIKV cases. Samples were obtained within five days from fever onset. To investigate if CHIKV was present in Mexico prior to October 2014, patients sampled between January and November 2014 principally from Chiapas and surrounding states (Campeche, Oaxaca, Tabasco and Veracruz) who met suspect case definition for dengue virus (DENV) infection (a patient with a non-specified fever who lives or who recently travelled to a region with reported dengue transmission)[49] but were negative for DENV infection, were submitted for CHIKV diagnosis. Molecular epidemiological studies of CHIKV infection were carried out on 25 patient specimens. To have a broad overview of viral diversity in areas of high transmission, specimens were included that were sampled in Southern Mexico (the region with most confirmed CHIKV cases during the sampling period of the study) and that were sampled at time points spanning December 2014 and August 2015. In addition, inclusion criteria included sample availability for sequencing and Ct values following RT-PCR diagnosis ≤ 32.

### Dengue diagnosis

Dengue diagnosis was done according to the algorithms and protocols issued by the InDRE[50]. In brief, serological assays for Dengue-specific IgM and IgG (Panbio, Korea) and for non-structural protein 1 (NS1; Bio-Rad, California) were done using capture ELISAs. Multiplex reverse-transcription quantitative PCR (qRT-PCR) was done, as described by Chien *et al*[51]) (primers in **S5 Table)**, on NS1 positive samples.

### CHIKV diagnosis

CHIKV diagnosis was done according to guidelines issued by the InDRE[49] and the Pan-American Health Organization (PAHO)[52]. Viral RNA was extracted from 200μL of patient serum using the QiAmp Viral RNA Extraction Kit (Qiagen, Hilden, Germany). Forward and reverse primers (CHIKV 6856 and CHIKV 6981, respectively) and a Carboxyfluorescein (FAM)-labelled probe (CHIKV 6919-FAM) were used as described by Lanciotti *et al*[53] **(S5 Table)**. The presence of CHIKV RNA was evaluated using the QuantiTect Probe RT-PCR kit (Qiagen) in a 25μL reaction using 12.5μL of 2x reverse transcription master mix, 0.25μL of QuantiTect RT mix, 0.25μL of each primer (1μM final concentration), probe 0.15μL (0.15μM final concentration), 6.6μL of water and 5μL de RNA. Using the Applied Biosystems 7500 Fast system (Applied Biosystems, Foster City, USA), reverse transcription was carried out at 50°C for 30 mins followed by 95°C for 15 minutes and 45 cycles of 95°C for 15 seconds and 56°C for 1 minute. As stated by the InDRE guidelines[49], Ct values ≤ 38 in duplicate wells were considered positive.

### CHIKV E1 sequencing and assembly

A 1044bp region of the CHIKV E1 gene was generated from extracted RNA (as described above) using primers COF1 and COR2 **(S5 Table)** and the QuantiTect Probe RT-PCR kit (Qiagen) with the conditions described above. For DNA sequencing, two overlapping amplicons (550 base pairs [bp] and 568bp) were then generated using modified primers COF1/CIR1 and CIF2/COR2 (**S5 Table**), that include adapter and barcode sequences for the Illumina sequencing. (Illumina, California). Both amplicons obtained for each patient were sequenced on the MiSeq platform (Illumina) with a 600 cycles V3 kit with a paired-end sequencing configuration to obtain 300bp paired end overlapping reads, following the manufacturer’s instructions. For each patient, the paired end reads for each amplicon (COF1 and CIF2 amplicons) were overlapped using FLASH v1.2.7[54] with default parameters and non-overlapping sequences were discarded. After reconstructing COF1 and CIF2 amplicons, identical nucleotide fragments were clustered, using CD-HIT v4.6.1[55] with -C 1. The output from the clustering process (.clust file) was transformed to a tab-separated list using the clstr2txt.pl script included in the CD-HIT suite and clusters with at least 50 amplicons, were considered. From each nucleotide cluster, the number of identical sequence amplicons forming the cluster was recorded. The COF1 and CIF2 amplicon sequences with the highest yield were also overlapped (74bp overlap) and aligned to the genome of the African prototype S27 CHIKV strain (accession number AF369024). Following a minimal sequence editing, the consensus sequence for the partial open reading frame for CHIKV E1 gene was identified (1,044bp, genome positions 10,264–11,308). All raw sequencing data was submitted to NCBI Bioproject ID PRJNA495608.

### Intra-patient variability analysis of CHIKV E1

For each nucleotide cluster, the representative sequence was translated using the program Transeq from the EMBOSS suite[56]. The translated representative sequences were also clustered at 100% of amino acid identity using CD-HIT (using -C 1 option). The sequence clusters represented an amino acid haplotype. Using the number of sequences in each nucleotide cluster that was previously recorded, the abundance for each amino acid cluster was calculated and normalized using the sequencing yield from the patient with the lowest yield as the normalization factor. A matrix with the normalized abundance of each haplotype and patient names was generated and visualized using the program Cluster v3.0 and Java TreeView v1.1.6r4, respectively. To calculate individual amino acid mutation frequencies, the number of reads forming all amino acid clusters containing a particular mutation was divided by the total number of reads forming all amino acid clusters in the patient.

### Phylogenetic analysis

To build the dataset used for the analysis, all available whole CHIKV genomes (334 genomes) plus all available E1 sequences that spanned the 1,044bp consensus fragment under study (384 sequences) were obtained from the NIAID Virus Pathogen Database and Analysis Resource[57] (ViPR; http://www.viprbrc.org/) and aligned together in ClustalW[56] with the 25 sequences generated in this study. An initial tree was built in PhyML version 3.0[58] under the General Time Reversible substitution model with a gamma distributed rate variation among sites (GTR+G), as suggested by the Smart Model Selection (SMS) online execution tool (http://www.atgc-montpellier.fr/sms/), with branch support estimated by the Approximate Likelihood Ratio Test (aLRT)-Shimodaira-Hasegawa-like (SH) procedure[59]. Due to our focus on evolution in the Asian lineage, we built our final dataset using E1 sequences from partial genomes only if they belonged to the Asian lineage (101 strains), as determined by the initial tree (aLRT = 1 for the monophyletic Asian lineage node). Sequences from all other lineages were from whole genomes. In addition, the following strains were removed due to suggestions of laboratory contamination, assembly error or high passage as suggested by the initial tree and previous studies[4,15]: Angola/M2022/1962, India/MH4/2000, India/ALSA-1/1986, India/STMWG01/2011, India/STMWG02/2011, Ross and S27. Finally, strains with missing information on sampling year were removed. The final dataset consisted of 420 strains: 12 West African, 17 non-epidemic ECSA, 142 IOL and 249 Asian strains.

The maximum liklihood (ML) phylogeny of the final dataset was made in PhyML using a starting tree generated in MEGA version 7.0.14[60]) with an O’nyong‘yong virus E1 sequence (strain IBH10964) inlcluded as an outgroup. Both starting tree and the PhyML analysis were done using the GTR+G evolutionary model described above and branch support in PhyML was estimated by the aLRT-SH procedure. For Bayesian Markov Chain Monte Carlo (MCMC) phylogenies, CHIKV lineages were analysed seperately in BEAST version 1.8.3[61]. Strains with missing sampling day were assigned the 15^th^ day of the month; missing sampling months and days were assigned as July 2^nd^. The temporal signal of the alignments was evaulated in TempEst v1.5[62] using ML trees generated in MEGA version 7.0.14 using the methods described above. BEAST analysis was done under the GTR+G nucleotide substitution model or under the Hasegawa-Kishino-Yano nucleotide substitution model with gamma distributed rate variation among sites (HKY+G), a lognormal relaxed clock and a Bayesian Skygrid coalescent model. Effective sample size scores of traces were evaluated in Tracer v1.6. Due to low ESS values under GTR+G (likely because of overparameterisation of the model) all subsequent analyses were done using the HKY+G substitution model. LogCombiner v1.8.3 was used to combine runs of fifty million MCMC states until convergence was reached and a maximum clade credibility (MCC) tree was determined in TreeAnnotator v1.8.3 from the sampling posterior after discarding 10% of states as burn-in. Nodes with posterior probabilities >0.9 were considered significant and evolutionary rates were considered significantly different if the 95% highest posterior density (HPD) intervals did not overlap. MCC trees were edited in FigTree v1.4.2.

## Supporting information

S1 Fig Root-to-tip divergence of CHIKV lineage phylogenies. Linear regression of the root-to-tip genetic distances (y axis) against sampling time (x axis) of five dataset alignments with a ‘best fit’ root position. Estimates of the rate of nucleotide substitution (the slope or gradient of the regression), point estimates of the time to most recent common ancestor (TMRCA) and the correlation coefficient (R^2^), are shown. ECSA, East Central Southern Africa lineage; IOL, Indian Ocean lineage; WAf, West African lineage.
(PDF)

S2 Fig Correlation between the number of haplotype variants in two E1 amplicons. Pearson’s correlation analysis between the number of variant haplotypes in each patient in amplicon COF1 and CIF2.
(PDF)

S1 Table Patient characteristics included in national passive CHIKV surveillance. (PDF)

S2 Table Characteristics of 25 CHIKV infected patients from 5 Mexican states. (PDF)

S3 Table The sequencing yield of paired-end reads and the percentage of overlapped reads for E1 amplicons. (PDF)

S4 Table Frequency of intrapatient amino acid mutations in two E1 amplicons and the viral haplotypes in which they occur. (PDF)

S5 Table Primers used for CHIKV and Dengue virus diagnosis and for sequencing of the CHIKV E1 gene. (PDF)

S6 Table Demographic and clinical characteristics of 25 sequenced samples. (PDF)

## References

[1] Caglioti C, Lalle E, Castilletti C, Carletti F, Capobianchi MR, Bordi L. Chikungunya virus infection: an overview. New Microbiol. 2013;36(3):211–27. 23912863

[2] Ross RW. The Newala epidemic. III. The virus: isolation, pathogenic properties and relationship to the epidemic. J Hyg (Lond). 1956;54(2):177–91. 13346078 10.1017/s0022172400044442 PMC2218030

[3] Solignat M, Gay B, Higgs S, Briant L, Devaux C. Replication cycle of chikungunya: a re-emerging arbovirus. Virology. 2009;393(2):183–97. 10.1016/j.virol.2009.07.024 19732931 10.1016/j.virol.2009.07.024 PMC2915564

[4] Volk SM, Chen R, Tsetsarkin KA, Adams AP, Garcia TI, Sall AA, et al Genome-Scale Phylogenetic Analyses of Chikungunya Virus Reveal Independent Emergences of Recent Epidemics and Various Evolutionary Rates. J Virol. 2010;84(13):6497–504. 10.1128/JVI.01603-09 20410280 10.1128/JVI.01603-09 PMC2903258

[5] Kariuki Njenga M, Nderitu L, Ledermann JP, Ndirangu A, Logue CH, Kelly CHL, et al Tracking epidemic Chikungunya virus into the Indian Ocean from East Africa. J Gen Virol. 2008 11;89(Pt 11):2754–60. 10.1099/vir.0.2008/005413-0 18931072 10.1099/vir.0.2008/005413-0 PMC3347796

[6] Weaver SC. Arrival of chikungunya virus in the new world: prospects for spread and impact on public health. PLoS Negl Trop Dis. 2014 6;8(6):e2921 10.1371/journal.pntd.0002921 24967777 10.1371/journal.pntd.0002921 PMC4072586

[7] Schuffenecker I, Iteman I, Michault A, Murri S, Frangeul L, Vaney MC, et al Genome microevolution of chikungunya viruses causing the Indian Ocean outbreak. PLoS Med. 2006;3(7):1058–70. 10.1371/journal.pmed.0030263 PMC1463904 16700631

[8] Niyas KP, Abraham R, Unnikrishnan RN, Mathew T, Nair S, Manakkadan A, et al Molecular characterization of Chikungunya virus isolates from clinical samples and adult Aedes albopictus mosquitoes emerged from larvae from Kerala, South India. Virol J. 2010;7(9 2009):189 10.1186/1743-422X-7-189 20704755 10.1186/1743-422X-7-189 PMC2928196

[9] Petersen LR, Powers AM. Chikungunya: epidemiology. F1000Research. 2016;5:1–8. 10.12688/f1000research.7171.1 PMC4754000 26918158

[10] Leparc-Goffart I, Nougairede A, Cassadou S, Prat C, de Lamballerie X. Chikungunya in the Americas. Lancet. 2014;383:514 10.1016/S0140-6736(14)60185-9 24506907 10.1016/S0140-6736(14)60185-9

[11] Nunes MRT, Faria NR, de Vasconcelos JM, Golding N, Kraemer MU, de Oliveira LF, et al Emergence and potential for spread of Chikungunya virus in Brazil. BMC Med. 2015;13(1):102. 25976325 10.1186/s12916-015-0348-x PMC4433093

[12] Díaz-Quiñonez JA, Escobar-Escamilla N, Ortíz-Alcántara J, Vázquez-Pichardo M, de la Luz Torres-Rodríguez M, Nuñez-León A, et al Identification of Asian genotype of chikungunya virus isolated in Mexico. Virus Genes. 2016;52(1):127–9. 10.1007/s11262-015-1275-9 26781948 10.1007/s11262-015-1275-9

[13] Stapleford KA, Moratorio G, Henningsson R, Chen R, Matheus S, Enfissi A, et al Whole-Genome Sequencing Analysis from the Chikungunya Virus Caribbean Outbreak Reveals Novel Evolutionary Genomic Elements. PLoS Negl Trop Dis. 2016;10(1). 10.1371/journal.pntd.0004402 PMC4726740 26807575

[14] Gobierno de los Estados Unidos Mexicanos-Presidencia de la Republica. Cuarto Informe de Gobierno 2015–2016. Anexo Estadístico. 2016.

[15] Chen R, Puri V, Fedorova N, Lin D, Hari KL, Jain R, et al Comprehensive Genome-Scale Phylogenetic Study Provides New Insights on the Global Expansion of Chikungunya Virus. J Virol. 2016;90(23):JVI.01166–16. 10.1128/JVI.01166-16 PMC5110187 27654297

[16] Tan K-K, Kristy A, Sy D, Tandoc AO, Khoo J-J, Sulaiman S, et al Independent Emergence of the Cosmopolitan Asian Chikungunya Virus, Philippines 2012. Sci Rep. 2015;1–11. 10.1038/srep12279 PMC5378875 26201250

[17] Sahadeo NSD, Allicock OM, De Salazar PM, Auguste AJ, Widen S, Olowokure B, et al Understanding the evolution and spread of chikungunya virus in the Americas using complete genome sequences. Virus Evol. 2017;3(1):vex010 10.1093/ve/vex010 28480053 10.1093/ve/vex010 PMC5413804

[18] Voss JE, Vaney M-C, Duquerroy S, Vonrhein C, Girard-Blanc C, Crublet E, et al Glycoprotein organization of Chikungunya virus particles revealed by X-ray crystallography. Nature. 2010;468(7324):709–12. 10.1038/nature09555 21124458 10.1038/nature09555

[19] Pan American Health Organization. No of cases of Chikungunya Fever in the Americas—Cumulative Cases (May 13, 2016) [Internet]. 2016.

[20] Carrera JP, Díaz Y, Denis B, Barahona de Mosca I, Rodriguez D, Cedeño I, et al Unusual pattern of chikungunya virus epidemic in the Americas, the Panamanian experience. PLoS Negl Trop Dis. 2017;11(2):1–23. 10.1371/journal.pntd.0005338 PMC5336303 28222127

[21] Secretaria de Salud. “Casos Confirmados de Enfermedad por Virus del Zika”, Semana Epidemiológica 44 del 2017 [Internet]. 2017.

[22] Musso D, Cao-Lormeau VM, Gubler DJ. Zika virus: following the path of dengue and chikungunya? Lancet. 2015;386(9990):243–4. 10.1016/S0140-6736(15)61273-9 26194519 10.1016/S0140-6736(15)61273-9

[23] Díaz-Quiñonez JA, Ortiz-Alcántara J, Fragoso-Fonseca DE, Garcés-Ayala F, Escobar-Escamilla N, Vázquez-Pichardo M, et al Complete genome sequences of chikungunya virus strains isolated in Mexico: first detection of imported and autochthonous cases. Genome Announc. 2015;3(3):e00300–15. 10.1128/genomeA.00300-15 25953170 10.1128/genomeA.00300-15 PMC4424286

[24] Díaz-Quiñonez JA, López-Martínez I, Torres-Longoria B, Vázquez-Pichardo M, Cruz-Ramírez E, Ramírez-González JE, et al Evidence of the presence of the Zika virus in Mexico since early 2015. Virus Genes. 2016;52(6):855–7. 10.1007/s11262-016-1384-0 27557815 10.1007/s11262-016-1384-0

[25] Faria NR, Azevedo R do S da S, Kraemer MUG, Souza R, Cunha MS, Hill SC, et al Zika virus in the Americas: Early epidemiological and genetic findings. Science. 2016;352(6283):aaf5036. 10.1126/science.aaf5036 PMC4918795 27013429

[26] Tan Y, Pickett BE, Shrivastava S, Gresh L, Balmaseda A, Amedeo P, et al Differing epidemiological dynamics of Chikungunya virus in the Americas during the 2014–2015 epidemic. PLoS Negl Trop Dis. 2018;12(7):1–23. 10.1371/journal.pntd.0006670 PMC6085065 30059496

[27] Venturi G, Di Luca M, Fortuna C, Elena Remoli M, Riccardo F, Severini F, et al Detection of a chikungunya outbreak in Central Italy Detection of a chikungunya outbreak in Central. Euro Surveill. 2017;22(39):1–4. 10.2807/1560-7917.ES.2017.22.39.17-00646 PMC5709953 29019306

[28] Lourenço-de-Oliveira R, Failloux A-B. High risk for chikungunya virus to initiate an enzootic sylvatic cycle in the tropical Americas. PLoS Negl Trop Dis. 2017;11(6):e0005698 10.1371/journal.pntd.0005698 28662031 10.1371/journal.pntd.0005698 PMC5507584

[29] Holmes EC, Dudas G, Rambaut A, Andersen KG. The evolution of Ebola virus: Insights from the 2013–2016 epidemic. Nature. 2016;538(7624):193–200. 10.1038/nature19790 27734858 10.1038/nature19790 PMC5580494

[30] Forrester NL, Coffey LL, Weaver SC. Arboviral bottlenecks and challenges to maintaining diversity and fitness during mosquito transmission. Viruses. 2014;6(10):3991–4004. 10.3390/v6103991 25341663 10.3390/v6103991 PMC4213574

[31] Abraham R, Manakkadan A, Mudaliar P, Joseph I, Sivakumar KC, Nair RR, et al Correlation of phylogenetic clade diversification and in vitro infectivity differences among Cosmopolitan genotype strains of Chikungunya virus. Infect Genet Evol. 2016;37:174–84. 10.1016/j.meegid.2015.11.019 26611825 10.1016/j.meegid.2015.11.019

[32] Wang C, Saborio S, Gresh L, Eswarappa M, Wu D, Fire A, et al Chikungunya virus sequences across the first epidemic in Nicaragua, 2014–2015. Am J Trop Med Hyg. 2016;94(2):400–3. 10.4269/ajtmh.15-0497 26643533 10.4269/ajtmh.15-0497 PMC4751928

[33] Vignuzzi M, Stone JK, Arnold JJ, Cameron CE, Andino R. Quasispecies diversity determines pathogenesis through cooperative interactions in a viral population. Nature. 2006;439(7074):344–8. 10.1038/nature04388 16327776 10.1038/nature04388 PMC1569948

[34] Coffey LL, Beeharry Y, Borderia a. V., Blanc H, Vignuzzi M. Arbovirus high fidelity variant loses fitness in mosquitoes and mice. Proc Natl Acad Sci. 2011;108(38):16038–43. 10.1073/pnas.1111650108 21896755 10.1073/pnas.1111650108 PMC3179076

[35] Rozen-Gagnon K, Stapleford KA, Mongelli V, Blanc H, Failloux AB, Saleh MC, et al Alphavirus Mutator Variants Present Host-Specific Defects and Attenuation in Mammalian and Insect Models. PLoS Pathog. 2014;10(1). 10.1371/journal.ppat.1003877 PMC3894214 24453971

[36] Rodriguez-Roche R, Blanc H, Bordería A V., Díaz G, Henningsson R, Gonzalez D, et al Increasing clinical severity during a dengue virus type 3 Cuban epidemic: deep sequencing of evolving viral populations. J Virol. 2016;90(2):JVI.02647–15. 10.1128/JVI.02647-15 PMC4836355 26889031

[37] Danis LR, Díaz GEE, Trujillo MKDC, Caballero SS, Sepulveda DJ, Malo GIR et al Clinical characterization of acute and convalescent illness of confirmed chikungunya cases from Chiapas, S. Mexico: A cross sectional study. PLoS One. 2017 10, 12 (10). 10.1371/journal.pone.0186923 PMC5655440 29065182

[38] Cigarroa TN, Blitvich BJ, Cetina TRC, Talavera ALG, Baak BCM, Torres COM et al Chikungunya Virus in febrile humans and aedes aegypri mosquitoes, Yucatan Mexico. Emerg Infect Dis. 2016 10, 22 (10): 1804–1807. 10.3201/eid2210.152087 27347760 10.3201/eid2210.152087 PMC5038406

[39] Rodriguez-Morales A, Gil-Restrepo AF, Ramírez-Jaramillo V, Montoya-Arias CP, Acevedo-Mendoza WF, Juan E B-A, et al Post-chikungunya chronic inflammatory rheumatism: results from a retrospective follow-up study of 283 adult and child cases in La Virginia, Risaralda, Colombia. F1000Research. 2016;5(5):360 doi: 10.12688/f1000research.8235.2 27081477 10.12688/f1000research.8235.1 PMC4813633

[40] Galán-Huerta KA, Martínez-Landeros E, Delgado-Gallegos JL, Caballero-Sosa S, Malo-García IR, Fernández-Salas I, et al Molecular and clinical characterization of Chikungunya virus infections in Southeast Mexico. Viruses. 2018;10(5):1–18. 10.3390/v10050248 PMC5977241 29747416

[41] Domingo E, Sheldon J, Perales C, Fitness V, Gain F, Equilibrium P, et al Viral Quasispecies Evolution. 2012;76(2):159–216. 10.1128/MMBR.05023-11 PMC3372249 22688811

[42] Aaskov J, Buzacott K, Thu HM, Lowry K, Holmes EC. Long-term transmission of defective RNA viruses in humans and Aedes mosquitoes. Science. 2006 1 13;311(5758):236–8. 10.1126/science.1115030 16410525 10.1126/science.1115030

[43] Forrester NL, Guerbois M, Adams AP, Liang X, Weaver SC. Analysis of Intrahost Variation in Venezuelan Equine Encephalitis Virus Reveals Repeated Deletions in the 6-Kilodalton Protein Gene. J Virol. 2011;85(17):8709–17. 10.1128/JVI.00165-11 21715498 10.1128/JVI.00165-11 PMC3165814

[44] Geller R, Domingo-Calap P, Cuevas JM, Rossolillo P, Negroni M, Sanjuán R. The external domains of the HIV-1 envelope are a mutational cold spot. Nat Commun. 2015;6:1–9. 10.1038/ncomms9571 PMC4687473 26450412

[45] Sun S, Xiang Y, Akahata W, Holdaway H, Pal P, Zhang X, et al Structural analyses at pseudo atomic resolution of Chikungunya virus and antibodies show mechanisms of neutralization. Elife. 2013;2013(2):1–27. 10.7554/eLife.00435 PMC3614025 23577234

[46] Jin J, Liss NM, Chen DH, Liao M, Fox JM, Shimak RM, et al Neutralizing Monoclonal Antibodies Block Chikungunya Virus Entry and Release by Targeting an Epitope Critical to Viral Pathogenesis. Cell Rep. 2015;13(11):2553–64. 10.1016/j.celrep.2015.11.043 26686638 10.1016/j.celrep.2015.11.043 PMC4720387

[47] Lum F-M, Teo T-H, Lee WWL, Kam Y-W, Rénia L, Ng LFP. An essential role of antibodies in the control of Chikungunya virus infection. J Immunol. 2013;190(12):6295–302. 10.4049/jimmunol.1300304 23670192 10.4049/jimmunol.1300304 PMC3677171

[48] Erasmus JH, Rossi SL, Weaver SC. Development of vaccines for chikungunya fever. J Infect Dis. 2016;214(Suppl 5):S488–96. 10.1093/infdis/jiw271 27920179 10.1093/infdis/jiw271 PMC5137239

[49] Dirección General de Epidemiología I. Lineamientos para la vigilancia epidemiológica y diagnóstico por laboratorio de fiebre chikungunya [Internet]. 2014.

[50] Instituto de Diagnóstico y Referencia Epidemiológicos. Lineamientos para la vigilancia epidemiológica de dengue por laboratorio. México; 2014. Chien L-J, 51.

[51] Chien L-J, Liao T-L, Shu P-Y, Huang J-H, Gubler DJ, Chang G-JJ. Development of real-time reverse transcriptase PCR assays to detect and serotype dengue viruses. J Clin Microbiol. 2006 4;44(4):1295–304. 10.1128/JCM.44.4.1295-1304.2006 16597854 10.1128/JCM.44.4.1295-1304.2006 PMC1448645

[52] Organización Panamericana de la Salud. Preparación y respuesta ante la eventual introducción del virus chikungunya en las Américas. Washington, D.C.; 2011.

[53] Lanciotti RS, Kosoy OL, Laven JJ, Panella AJ, Velez JO, Lambert AJ, et al Chikungunya virus in US travelers returning from India, 2006. Emerg Infect Dis. 2007 5;13(5):764–7. 10.3201/eid1305.070015 17553261 10.3201/eid1305.070015 PMC2738459

[54] Salzberg SL. FLASH: fast length adjustment of short reads to improve genome assemblies Tanja Mago c. 2011;27(21):2957–63. 10.1093/bioinformatics/btr507 PMC3198573 21903629

[55] Li W, Godzik A. Cd-hit: a fast program for clustering and comparing large sets of protein or nucleotide sequences. Bioinformatics. 2006 7 1;22(13):1658–9. 10.1093/bioinformatics/btl158 16731699 10.1093/bioinformatics/btl158

[56] Li W, Cowley A, Uludag M, Gur T, McWilliam H, Squizzato S, et al The EMBL-EBI bioinformatics web and programmatic tools framework. Nucleic Acids Res. 2015;43(W1):W580–4. 10.1093/nar/gkv279 25845596 10.1093/nar/gkv279 PMC4489272

[57] Pickett BE, Sadat EL, Zhang Y, Noronha JM, Squires RB, Hunt V, et al ViPR: An open bioinformatics database and analysis resource for virology research. Nucleic Acids Res. 2012;40(D1):593–8. 10.1093/nar/gkr859 PMC3245011 22006842

[58] Guindon S, Dufayard J-F, Lefort V, Anisimova M, Hordijk W, Gascuel O. New algorithms and methods to estimate maximum-likelihood phylogenies: assessing the performance of PhyML 3.0. Syst Biol. 2010 5;59(3):307–21. 10.1093/sysbio/syq010 20525638 10.1093/sysbio/syq010

[59] Anisimova M, Gascuel O. Approximate likelihood-ratio test for branches: A fast, accurate, and powerful alternative. Syst Biol. 2006 8;55(4):539–52. 10.1080/10635150600755453 16785212 10.1080/10635150600755453

[60] Kumar S, Stecher G, Tamura K. MEGA7: Molecular Evolutionary Genetics Analysis Version 7.0 for Bigger Datasets. Mol Biol Evol. 2016 7;33(7):1870–4. 10.1093/molbev/msw054 27004904 10.1093/molbev/msw054 PMC8210823

[61] Drummond AJ, Rambaut A. BEAST: Bayesian evolutionary analysis by sampling trees. BMC Evol Biol. 2007 1;7:214 10.1186/1471-2148-7-214 17996036 10.1186/1471-2148-7-214 PMC2247476

[62] Rambaut A, Lam TT, Max Carvalho L, Pybus OG. Exploring the temporal structure of heterochronous sequences using TempEst (formerly Path-O-Gen). Virus Evol. 2016;2(1):vew007 10.1093/ve/vew007 27774300 10.1093/ve/vew007 PMC4989882

