# Supplementary material for: Evolutionary analysis of the Chikungunya virus epidemic in Mexico reveals intra-host mutational hotspots in the E1 protein
Source: PLoS One. 2018 Dec 14;13(12):e0209292. doi: 10.1371/journal.pone.0209292 (PMC6294367; doi:10.1371/journal.pone.0209292)
Supplement: S1 Fig — Linear regression of the root-to-tip genetic distances (y axis) against sampling time (x axis) of five dataset alignments with a ‘best fit’ root position. Estimates of the rate of nucleotide substitution (the slope or gradient of the regression), point estimates of the time to most recent common ancestor (TMRCA) and the correlation coefficient (R2), are shown. ECSA, East Central Southern Africa lineage; IOL, Indian Ocean lineage; WAf, West African lineage. (PDF) [file pone.0209292.s001.pdf]

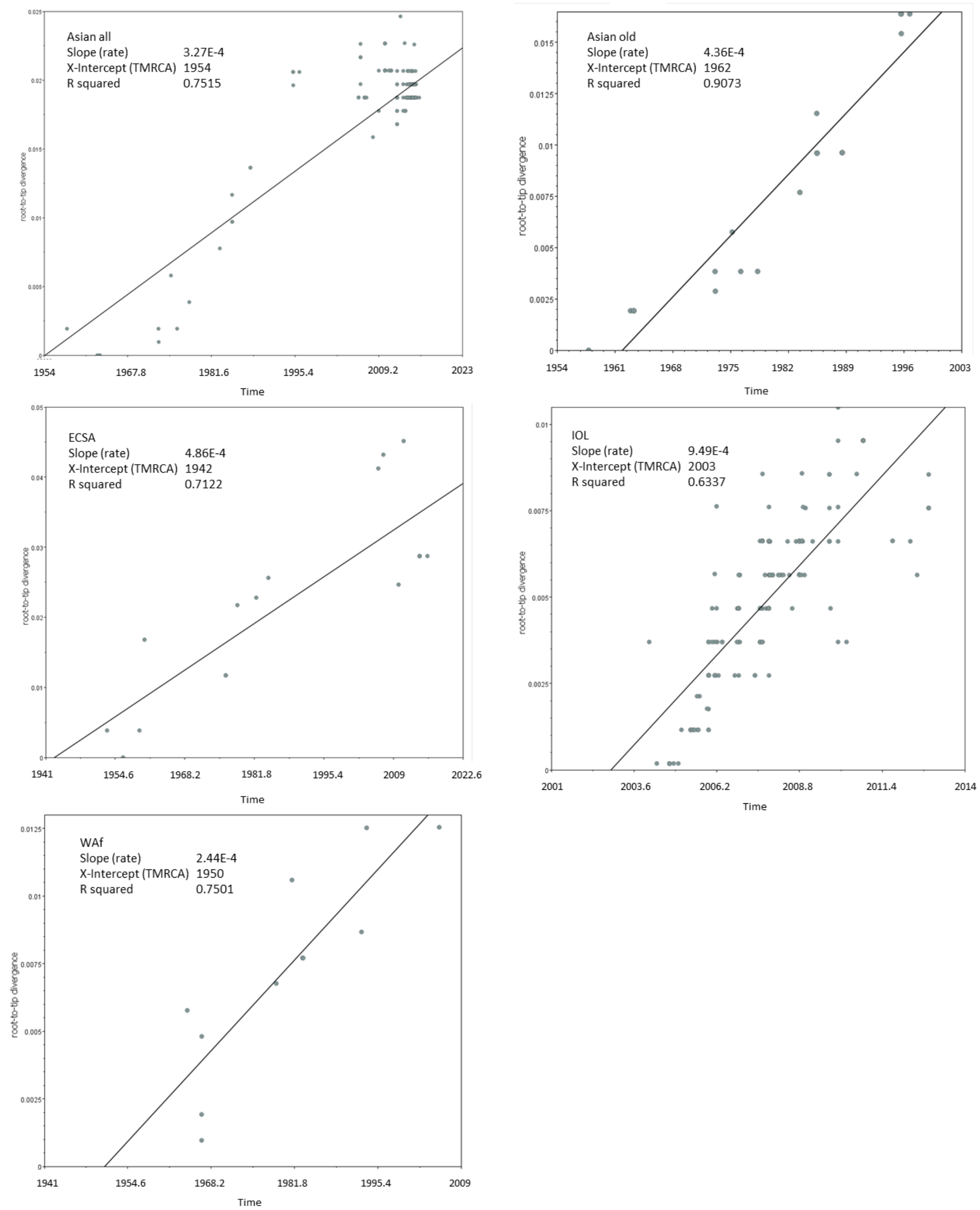

S1 Fig. Root-to-tip divergence of CHIKV lineage phylogenies. Linear regression of the root-to-tip genetic distances (y axis) against sampling time (x axis) of five dataset alignments with a ‘best fit’ root position. Estimates of the rate of nucleotide substitution (the slope or gradient of the regression), point estimates of the time to most recent common ancestor (TMRCA) and the correlation coefficient (R^2), are shown. ECSA, East Central Southern Africa lineage; IOL, Indian Ocean lineage; WAf, West African lineage.
