# Supplementary material for: Evolutionary analysis of the Chikungunya virus epidemic in Mexico reveals intra-host mutational hotspots in the E1 protein
Source: PLoS One. 2018 Dec 14;13(12):e0209292. doi: 10.1371/journal.pone.0209292 (PMC6294367; doi:10.1371/journal.pone.0209292)
Supplement: S2 Fig — Pearson’s correlation analysis between the number of variant haplotypes in each patient in amplicon COF1 and CIF2. (PDF) [file pone.0209292.s002.pdf]

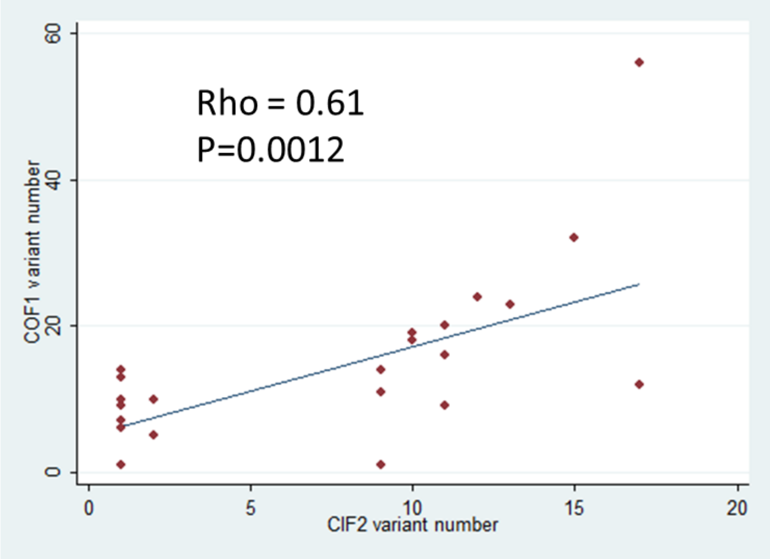

S2 Fig. Correlation between the number of haplotype variants in two E1 amplicons.

Pearson's correlation analysis between the number of variant haplotypes in each patient in amplicon COF1 and CIF2**.**
