# Supplementary material for: Evolutionary analysis of the Chikungunya virus epidemic in Mexico reveals intra-host mutational hotspots in the E1 protein
Source: PLoS One. 2018 Dec 14;13(12):e0209292. doi: 10.1371/journal.pone.0209292 (PMC6294367; doi:10.1371/journal.pone.0209292)
Supplement: S1 Table — (PDF) [file pone.0209292.s003.pdf]

S1 Table. Patient characteristics included in national passive CHIKV surveillance

Parameter	N (total 5266)	RT-qPCR CHIKV Positive	RT-qPCR-CHIKV Negative	P value[a]
**Age[b]**				
≤ 17	802	454	348	< 0.0001
18-29	1218	586	632	
30-39	1124	546	578	
40-49	862	443	419	
50-59	574	311	263	
>60	446	278	168	
**Gender[c]**				
Female	3271	1679	1592	NS
Male	1968	1046	922	
**Location[d]**				
South	4041	2082	1959	<0.0001
Centre	889	516	373	
North	147	59	88	

[a]Chi Square Test of dependency between being CHIKV positive and age, gender and location
[b]N=5026 included in the analysis as 240 patients had missing data
[c]N=5239 included in the analysis as 27 patients had missing data
[d]N=5077 included in the analysis as 189 patients had missing data
