# Supplementary material for: Evolutionary analysis of the Chikungunya virus epidemic in Mexico reveals intra-host mutational hotspots in the E1 protein
Source: PLoS One. 2018 Dec 14;13(12):e0209292. doi: 10.1371/journal.pone.0209292 (PMC6294367; doi:10.1371/journal.pone.0209292)
Supplement: S2 Table — (PDF) [file pone.0209292.s004.pdf]

S2 Table. Characteristics of 25 CHIKV infected patients from 5 Mexican states

Sample	Age, y	Gender	State	Sampling date	Accession number
Veracruz_1	14	M	Veracruz (South)	29/12/2014	SRX4824663
Colima_2	60	F	Colima	22/04/2015	SRX4824662
Guerrero_3	41	F	Guerrero	04/02/2015	SRX4824661
Veracruz_4	62	M	Veracruz (North)	29/12/2014	SRX4824660
Colima_5	74	F	Colima	21/04/2015	SRX4824659
Chiapas_6	20	F	Chiapas	20/01/2015	SRX4824658
Chiapas_7	49	F	Chiapas	23/12/2014	SRX4824657
Chiapas_8	20	F	Chiapas	13/02/2015	SRX4824656
Guerrero_9	59	M	Guerrero	30/01/2015	SRX4824655
Colima_10	83	M	Colima	21/04/2015	SRX4824654
Oaxaca_11	38	F	Oaxaca	16/02/2015	SRX4824649
Oaxaca_12	67	M	Oaxaca	05/03/2015	SRX4824648
Colima_13	28	M	Colima	20/04/2015	SRX4824647
Colima_14	10	F	Chiapas	21/04/2015	SRX4824646
Guerrero_15	63	F	Guerrero	20/04/2015	SRX4824653
Guerrero_16	38	M	Guerrero	05/03/2015	SRX4824652
Chiapas_17	61	F	Chiapas	06/02/2015	SRX4824651
Guerrero_18	8	F	Guerrero	23/04/2015	SRX4824650
Oaxaca_19	30	F	Oaxaca	24/06/2015	SRX4824645
Veracruz_20	57	M	Veracruz (North)	20/07/2015	SRX4824644
Veracruz_21	51	M	Veracruz (South)	05/08/2015	SRX4824667
Oaxaca_22	36	M	Oaxaca	06/08/2015	SRX4824668
Colima_23	43	M	Colima	10/08/2015	SRX4824665
Veracruz_24	48	F	Veracruz (North)	29/12/2014	SRX4824666
Oaxaca_25	7	M	Oaxaca	22/04/2015	SRX4824664

y, years; M, male; F, female
