# Supplementary material for: Evolutionary analysis of the Chikungunya virus epidemic in Mexico reveals intra-host mutational hotspots in the E1 protein
Source: PLoS One. 2018 Dec 14;13(12):e0209292. doi: 10.1371/journal.pone.0209292 (PMC6294367; doi:10.1371/journal.pone.0209292)
Supplement: S3 Table — (PDF) [file pone.0209292.s005.pdf]

S3 Table. The sequencing yield of paired-end reads and the percentage of overlapped reads for E1 amplicons.

Patient	COF1 amplicon			CIF2 amplicon		
	Total PE reads	Overlapped (%)	Norm	Total PE reads	Overlapped (%)	Norm
Veracruz_1	37356	24015 (64.29%)	4.73389	17411	9040 (51.92%)	3.70948
Colima_2	77932	53791 (69.02%)	10.6034	27919	15911 (56.99%)	6.52893
Guerrero_3	58504	37275 (63.71%)	7.34772	54937	30942 (56.32%)	12.6968
Veracruz_4	9676	5073 (52.43%)	1	5430	2437 (44.88%)	1
Colima_5	27633	18560 (67.17%)	3.65858	20665	11878 (57.48%)	4.87403
Chiapas_6	30290	19389 (64.01%)	3.822	19890	10768 (54.14%)	4.41855
Chiapas_7	113840	77756 (68.30%)	15.3274	67312	42996 (63.88%)	17.643
Chiapas_8	40576	28131 (69.33%)	5.54524	54510	34947 (64.11%)	14.3402
Guerrero_9	120707	76572 (63.44%)	15.094	59757	37216 (62.28%)	15.2712
Colima_10	164072	104221 (63.52%)	20.5443	75162	48836 (64.97%)	20.0394
Oaxaca_11	136085	87379 (64.21%)	17.2243	66466	43931 (66.10%)	18.0267
Oaxaca_12	106852	70260 (65.75%)	13.8498	46335	29267 (63.16%)	12.0094
Colima_13	93593	64029 (68.41%)	12.6215	86531	55401 (64.02%)	22.7333
Chiapas_14	113926	75646 (66.40%)	14.9115	48333	31153 (64.45%)	12.7833
Guerrero_15	77956	52429 (67.25%)	10.3349	67853	41789 (61.59%)	17.1477
Guerrero_16	84200	55755 (66.22%)	10.9905	53952	34749 (64.41%)	14.2589
Chiapas_17	74434	52418 (70.42%)	10.3327	41756	27182 (65.10%)	11.1539
Guerrero_18	47912	32749 (68.35%)	6.45555	44589	27939 (62.66%)	11.4645
Oaxaca_19	63464	35357 (55.71%)	6.96964	40497	18688 (46.15%)	7.66844
Veracruz_20	56911	31560 (55.46%)	6.22117	31119	13722 (44.10%)	5.63069
Veracruz_21	86128	45639 (52.99%)	8.99645	32854	14072 (42.83%)	5.77431
Oaxaca_22	97643	56060 (57.41%)	11.0507	35973	16950 (47.12%)	6.95527
Colima_23	49864	27490 (55.13%)	5.41888	23961	11430 (47.70%)	4.69019
Veracruz_24	65942	36476 (55.32%)	7.19022	31971	14309 (44.76%)	5.87156
Oaxaca_25	442131	318709 (72.08%)	62.8246	145483	92816 (63.80%)	38.0862

Norm: Normalization factor for each sample according to the patient with the lowest yield; PE: paired-end
