# Supplementary material for: Evolutionary analysis of the Chikungunya virus epidemic in Mexico reveals intra-host mutational hotspots in the E1 protein
Source: PLoS One. 2018 Dec 14;13(12):e0209292. doi: 10.1371/journal.pone.0209292 (PMC6294367; doi:10.1371/journal.pone.0209292)
Supplement: S4 Table — (PDF) [file pone.0209292.s006.pdf]

S4 Table. Frequency of intrapatient amino acid mutations in two E1 amplicons and the viral haplotypes in which they occur

Amino acid mutation*	Veracruz_1	Colima_2	Guerrero_3	Veracruz_4	Colima_5	Chiapas_6	Chiapas_7	Chiapas_8	Guerrero_9	Colima_10	Oaxaca_11	Oaxaca_12	Colima_13	Chiapas_14	Guerrero_15	Guerrero_16	Chiapas_17	Guerrero_18	Oaxaca_19	Veracruz_20	Veracruz_21	Oaxaca_22	Colima_23	Veracruz_24	Oaxaca_25	Haplotype
C96F									0.003	0.003	0.003														0.003	COF 1_31
I177F																									0.002	COF 1_42
Y185H							0.003			0.002	0.002														0.002	COF 1_23
Y189D																									0.002	COF 1_50
P191T	0.022	0.029	0.031			0.026	0.027	0.031	0.029	0.025	0.025	0.024	0.025	0.025	0.029	0.025	0.028	0.03	0.027	0.027	0.04	0.03	0.023	0.038	0.032	COF 1_5, COF 63_1, COF 1_47, COF 1_43
P191R																									0.002	COF 1_44
P191H																									0.002	COF 1_49
P191S																									0.002	COF 1_54
P191A		0.006					0.013		0.011	0.01	0.011	0.01	0.011	0.011	0.01	0.01	0.011					0.014			0.011	COF 1_10, COF 1_67, COF 1_41
F192S										0.002															0.002	COF 1_29
F192C	0.035	0.037	0.034		0.026	0.026	0.029	0.035	0.028	0.026	0.026	0.033	0.026	0.032	0.032	0.03	0.03	0.036	0.043	0.028	0.041	0.034	0.032	0.041	0.029	COF 1_3, COF 1_58, COF 1_46, COF 1_43
F192V	0.041	0.051	0.054		0.071	0.042	0.046	0.047	0.047	0.048	0.048	0.05	0.044	0.045	0.051	0.049	0.049	0.056	0.066	0.068	0.064	0.057	0.073	0.064	0.06	COF 1_2, COF 1_22, COF 1_66, COF 1_52, COF 1_47, COF 1_41, COF 1_51, COF 1_40, COF 1_36
F192I																									0.002	COF 1_48
F192Y										0.002															0.002	COF 1_28
F192L							0.003		0.003	0.003	0.003	0.003													0.004	COF 1_19
G193D																									0.002	COF 1_35
A194G										0.002	0.002														0.003	COF 1_32
A194E							0.009		0.01	0.009	0.01	0.006	0.011	0.009	0.011	0.009	0.005					0.007			0.007	COF 1_11, COF 1_65
G195R																									0.002	COF 1_37
R196S										0.002															0.002	COF 1_30
R196G																									0.002	COF 1_56
P197T							0.005		0.005	0.006	0.004	0.005		0.005	0.006	0.004	0.004								0.005	COF 1_12
P197Q		0.021	0.013				0.02	0.015	0.018	0.017	0.018	0.021	0.021	0.02	0.018	0.019	0.017	0.017	0.017	0.016	0.015	0.023		0.015	0.019	COF 1_7, COF 1_60, COF 1_51
G198V											0.002														0.002	COF 1_34
Q199K																									0.002	COF 1_53
Q199H							0.006		0.007	0.007	0.007	0.008		0.007	0.007	0.008	0.006	0.009			0.011	0.008			0.007	COF 1_13
F200I																									0.002	COF 1_55
F200V	0.027	0.034	0.038		0.028	0.03	0.037	0.041	0.036	0.035	0.038	0.034	0.03	0.032	0.038	0.034	0.033	0.038	0.045	0.046	0.046	0.038	0.032	0.045	0.037	COF 1_4, COF 1_22, COF 1_59, COF 1_46, COF 1_38
F200C		0.02	0.013			0.023	0.018	0.015	0.016	0.016	0.015	0.019	0.018	0.019	0.017	0.018	0.019	0.017	0.015	0.017	0.015	0.014		0.014	0.019	COF 1_8, COF 1_64, COF 1_52
F200L							0.003			0.005	0.002														0.006	COF 1_21
D202E																									0.002	COF 1_45
I203S		0.012	0.012				0.016	0.013	0.014	0.015	0.013	0.015	0.013	0.015	0.016	0.015	0.016	0.011	0.015	0.017	0.028	0.021	0.017	0.015	0.014	COF 1_9, COF 1_57, COF 1_36
I203T							0.003		0.003	0.003	0.003	0.003		0.004											0.003	COF 1_17
Q204L							0.006		0.008	0.007	0.009	0.008		0.007	0.008	0.007	0.008	0.01				0.008			0.008	COF 1_14
Q204H							0.003		0.005	0.003	0.004	0.004		0.004			0.004		0.011						0.004	COF 1_20

Q204P							0.003		0.003	0.003	0.003	0.003		0.004		0.004									0.003	COF 1_16
S205R	0.026	0.026	0.023		0.027	0.028	0.032	0.023	0.033	0.031	0.029	0.029	0.03	0.037	0.032	0.032	0.025	0.026	0.024	0.032	0.03	0.042	0.029	0.027	0.031	COF 1_6, COF 1_62, COF 1_40, COF 1_38
S205G										0.002															0.001	COF 1_26
**T207M**													0.967													COF 1_61, COF 1_66, COF 1_58, COF 1_67, COF 1_59, COF 1_63, COF 1_64, COF 1_60, COF 1_65, COF 1_62, COF 1_57
E209A							0.003		0.003	0.002	0.003			0.003											0.003	COF 1_18
S210R							0.003		0.003	0.003	0.003			0.003		0.004									0.004	COF 1_15
Y214X										0.002															0.002	COF 1_27
Q218P										0.002		0.003													0.001	COF 1_25
Q218H											0.002														0.001	COF 1_33
L219R							0.003			0.002															0.002	COF 1_24
V220G																									0.002	COF 1_39
**V291I**															0.692											CIF 2_13
I339F		0.175	0.101		0.192		0.054	0.048	0.047	0.044	0.042	0.055	0.059	0.05		0.052	0.073	0.06							0.043	CIF 2_2
N349K										0.009			0.009												0.006	CIF 2_16
S350A			0.064				0.045	0.05	0.05	0.051	0.049	0.051	0.045	0.045		0.048	0.045	0.052							0.05	CIF 2_4
L352W													0.009												0.008	CIF 2_17
Q353K							0.03	0.032	0.032	0.025	0.028	0.027	0.024	0.03		0.026	0.027	0.03							0.027	CIF 2_7
Q353P							0.014	0.014		0.012	0.013		0.01			0.017									0.01	CIF 2_11
I354T									0.014	0.012	0.012		0.01												0.008	CIF 2_12
I354S							0.024	0.022	0.029	0.025	0.028	0.027	0.026	0.023		0.025	0.029	0.031							0.022	CIF 2_6
I354L							0.019	0.016	0.016	0.017	0.018	0.021	0.017	0.019		0.02									0.012	CIF 2_10
S355P										0.01	0.011		0.01												0.011	CIF 2_15
S355A							0.023	0.027	0.027	0.021	0.021	0.019	0.022	0.025		0.023	0.022	0.025							0.02	CIF 2_9
F356C			0.052				0.045	0.048	0.058	0.048	0.049	0.054	0.047	0.052		0.047	0.057	0.055							0.052	CIF 2_3
F356S							0.018	0.023	0.02	0.02	0.023	0.025	0.017	0.021		0.014	0.022	0.022							0.016	CIF 2_8
F356L							0.033	0.038	0.033	0.035	0.036	0.035	0.032	0.034		0.037	0.041	0.034							0.031	CIF 2_5
T358P							0.011			0.009			0.009												0.007	CIF 2_14
S371A													0.009												0.008	CIF 2_18

*Position relative to start of E1 protein. Mutations present in the consensus sequence are in bold.
