# Supplementary material for: Evolutionary analysis of the Chikungunya virus epidemic in Mexico reveals intra-host mutational hotspots in the E1 protein
Source: PLoS One. 2018 Dec 14;13(12):e0209292. doi: 10.1371/journal.pone.0209292 (PMC6294367; doi:10.1371/journal.pone.0209292)
Supplement: S5 Table — (PDF) [file pone.0209292.s007.pdf]

S5 Table. Primers used for CHIKV and Dengue virus diagnosis and for sequencing of the CHIKV E1 gene

Name	Sequence 5'-3'	Reference
**Chikungunya diagnosis**		
**CHIKV 6856**	TCA CTC CCT GTT GGA CTT GAT AGA	Lanciotti et al
**CHIKV 6981**	TTG ACG AAC AGA GTT AGG AAC ATA CC	Lanciotti et al
**CHIKV 6919**	FAM AGG TAC GCG CTT CAA GTT CGG CG BHQ-2	Lanciotti et al
**Dengue diagnosis**		
**mFU1**	TAC AAC ATG ATG GGA AAG CGA GAG AAA AA	Chien et al
**CFD2**	GTG TCC CAG CCG GCG GTG TCA TCA GC	Chien et al
**D1**	FAM TCA GAG ACA TAT CAA AGA TTC CAG GGG G BHQ-1	Chien et al
**D2**	CAL FLUOR RED 610 AAG AGA CGT GAG CAG GAA GGA AGG GGG AGC BHQ-2	Chien et al
**D3**	QUASAR 670 TGA GAG ATA TTT CCA AGA TAC CCG GAG GAG BHQ-2	Chien et al
**D4**	HEX TGG AGG AGA TAG ACA AGA AGG ATG GAG ACC BHQ-1	Chien et al
**CHIKV E1amplification and sequencing**		
**COF1**	GGC GCC TAC TGC TTC TGC G	This study
**CIR1**	CT ACG GCG CAG TTC ATC GCT C	This study
**CIF2**	GTC GCT GCA GCA CAC AGC AC	This study
**COR2**	CCT GCT AAA CGA CAC GCA TAG CA	This study

References

Lanciotti, R. S. *et al.* Chikungunya virus in US travelers returning from India, 2006. *Emerging infectious diseases* **13,** 764–7 (2007).

Chien, L.J. *et al.* Development of real-time reverse transcriptase PCR assays to detect and serotype dengue viruses. *Journal of clinical microbiology* **44,** 1295–304 (2006).
