# Supplementary material for: Evolutionary analysis of the Chikungunya virus epidemic in Mexico reveals intra-host mutational hotspots in the E1 protein
Source: PLoS One. 2018 Dec 14;13(12):e0209292. doi: 10.1371/journal.pone.0209292 (PMC6294367; doi:10.1371/journal.pone.0209292)
Supplement: S6 Table — (PDF) [file pone.0209292.s008.pdf]

S6. Demographic and clinical characteristics of 25 sequencing samples

Parameter	Number	Frecuency (%)
**Gender**		
Female	12	48
Male	13	52
**Location**		
South	19	76
Centre	6	24
North	0	0
**Symptoms**		
Fever	25	100
Myalgia	24	92
Arthralgias	24	92
Headache	20	80
Chill	20	80
Arthritis	19	76
Retro-ocular pain	15	60
Exanthema	7	28
Abdominal pain	5	20
Fatigue	3	12
Conjunctivitis	2	8
Splenomegaly	2	8
Threw up	2	8
Diarrhea	2	8
Cough	1	4
Jaundice	1	4
Hepatomegaly	1	4
Decreased visual acuity	1	4
Hemorrhage	1	4
